# Spatial isotope tracing maps compartmentalized nutrient use across the adult fruit fly

**DOI:** 10.64898/2026.10.06.757091

**Published:** 2026-10-20

**Authors:** Sun Jin Moon, Kerui Huang, Jenna L. E. Blum, Connie New, Yooha Park, Yuchen Chen, Hiroshi Nishida, Veronika Pister, Md Ausrafuggaman Nahid, Yanhui Hu, Jason Tennessen, Ernest Fraenkel, John M. Asara, Shawn M. Davidson, Norbert Perrimon

## Abstract

*Drosophila melanogaster* is a powerful model for studying systemic metabolism. Although metabolism is compartmentalized across tissues, prior work in adult *Drosophila* has mostly measured it in dissected tissues rather than *in situ* , limiting the spatial information that can be gained. Here, we combine whole-fly massspectrometry imaging with stable-isotope tracing and flux modeling to quantify metabolic activities across the adult fruit fly in a spatially resolved manner. Across 68 tracer-fed Oregon-R female flies and 187,709 pixels, we map 81 metabolites onto eight annotated regions, including muscle, fat body, gut, and nervous system. Glucose labeling and fitted relative fluxes indicate higher glycolytic and tricarboxylic acid cycle turnover in thoracic muscle than in abdominal fat body; proline labels the nervous system and eye; glutamine the abdominal reproductive system and gut. Alanine carries glucose-derived carbon through the hemolymph and transfers nitrogen into glutamate. In flies bearing yorkie-driven gut tumors, alanine contribution to malate rises in gut and abdomen, and reducing gut alanine uptake lowers tumor burden. Together, this work establishes a framework for quantitative spatial metabolic analysis, showing compartmentalized nutrient use across the adult fruit fly.

